# Freshwater snails as the intermediate host of trematodes in Iran: a systematic review

**DOI:** 10.4178/epih.e2019001

**Published:** 2019-01-07

**Authors:** Samira Dodangeh, Ahmad Daryani, Mehdi Sharif, Shirzad Gholami, Elham Kialashaki, Mahmood Moosazadeh, Shahabeddin Sarvi

**Affiliations:** 1 Student Research Committee, Mazandaran University of Medical Sciences, Sari, Iran; 2 Department of Medical Parasitology and Mycology, Toxoplasmosis Research Center, Mazandaran University of Medical Sciences, Sari, Iran; 3 Health Sciences Research Center, Addiction Institute, Mazandaran University of Medical Sciences, Sari, Iran

**Keywords:** Meta-analysis, Prevalence, Snail, Trematodes, Iran

## Abstract

Freshwater snails, as the first intermediate hosts of trematodes, can cause health hazards in animals and humans. Recently, the World Health Organization has included Iran in a list of 6 countries known to have serious problems with fascioliasis. In addition, cercarial dermatitis is a job-related disease that is seen often in paddy workers, agricultural labourers, and fishermen in Iran, particularly in Mazandaran Province. Many studies have been conducted in Iran to survey larval trematodes in freshwater snails. However, to the best of our knowledge, no comprehensive data exist regarding infections in gastropods. Therefore, the aim of the present study was to estimate the types and prevalence of cercarial infections in snails in Iran. Electronic English-language and Persian-language databases were searched to identify 24 published articles reporting the prevalence of trematode infections in snails (9 species from 6 families) in various provinces of Iran. In total, 4.4% of gastropods were infected with the larval stages of trematodes. According to the studies reviewed in this meta-analysis‚ the highest infection prevalence was found in *Radix auricularia* (9.9%). Twelve larval species of trematodes were identified, and the highest prevalence of cercariae was found for Echinostomatidae cercariae (4.3%). Among the provinces explored, West Azerbaijan had the highest prevalence of infected snails (16.9%). The presence of trematodes in snails could pose a serious health problem in Iran. Thus, further studies are necessary to characterize these infections in other provinces.

## INTRODUCTION

Trematode infections pose serious risks to the health of their vertebrate hosts, including humans and livestock, and can adversely affect both agriculture and the economy [1]. Digenetic trematodes have a complicated life cycle that is initiated in their first intermediate hosts, such as freshwater snails, which are widespread in water sources in most geographical regions; the larval stages, such as sporocysts, rediae, and cercariae, develop within snails [2,3]. Therefore, the extent of human infections is primarily related to the rate of exposure to infective larvae. According to study of Doughty [4] has been reported that most infected people show no overt symptoms of disease. Instead, significant disease is mainly observed in a small subset of people with a heavy burden of trematodes. The distribution of these flukes depends on the availability of intermediate hosts (various snail species).

Three human schistosome species, *Schistosoma mansoni, Schistosoma japonicum*, and *Schistosoma haematobium*, are responsible for 200 million infections annually. In addition, cercarial dermatitis is a disease caused by other species of schistosomes, which parasitize mammals (mostly ruminants) and aquatic birds (especially the Anatidae family) [4]. Cercarial dermatitis is a job-related disease that is seen often in paddy workers, agricultural labourers, and fishermen in Iran, particularly in Mazandaran Province [5]. *Fasciola hepatica* occurs widely in sheep-rearing and cattle-rearing areas worldwide, causing severe morbidity and mortality. In Asia, human infections are mainly observed in Iran, and to a lesser extent in Vietnam. The World Health Organization has recently included Iran in a list of 6 countries that are known to have significant problems with fascioliasis [6]. *Opisthorchis viverrini* and *Clonorchis sinensis* are common liver flukes found in fish-eating mammals, especially cats and dogs, although humans can become infected by consuming undercooked freshwater fish containing metacercariae of these parasites. *Fasciolopsis buski*, the giant intestinal fluke, is the etiological agent of fasciolopsiasis in many mammals, particularly humans. Fasciolopsiasis occurs through the consumption of raw or undercooked aquatic plants containing the metacercariae. *Paragonimus westermani*, a lung fluke, has a wide variety of mammalian hosts, and can also infect humans who ingest insufficiently cooked crabs or crayfish contaminated with the encysted parasite [4].

The dynamics of ecosystems containing snails should be monitored in multiple areas, so that knowledge about the distributions of both the snail population and parasitic diseases in those areas may help to control the snail population, thereby improving community health.

Approximately 350 snail species are known to be of probable medical or veterinary importance. Among the intermediate hosts of trematodes, the species belonging the genera *Biomphalaria, Bulinus* (water), and *Oncomelania* (amphibious) are important in the transmission of human schistosomes. In addition, the most important intermediate hosts of liver flukes are members of the genus *Lymnaea*, which may be either aquatic or amphibious [7].

According to the available resources, various species of snails are found in different parts of Iran; but the fauna of Iran and relevant parasitic infections have not been extensively examined, unlike in other parts of the world [8].

Many different types of cercariae have been identified. For instance, Anucherngchai et al. [9] reported 9 types of cercarial infections—cercariae, furcocercous cercariae, echinostome cercariae, monostome cercariae, megarulous cercariae, parapleurolophocercous cercariae, pleurolophocercous cercariae, xiphidiocercariae, and virgulate cercariae—in freshwater gastropods from 10 provinces in the Chao-Phraya Basin.

Thus, collecting data on the prevalence of infections with different cercariae in snails is essential for estimating the risk of parasitic diseases in different parts of Iran. In recent years, some studies have been carried out on trematode infections in snails in Iran, but further research is necessary to understand these infections more completely. No relevant systematic review has been conducted about the prevalence of these infections in Iran. We therefore considered it necessary to summarize all available studies on such infections in freshwater snails in the form of a review. The aim of the present study was to estimate the types and prevalence of cercarial infections in snails in Iran. Moreover, this review provides easy access to the available literature and also considers the gaps left by previous studies, which would be beneficial for future researchers.

## MATERIALS AND METHODS

This review was conducted in accordance with the PRISMA (Preferred Reporting Items for Systematic Reviews and Meta-Analyses) guideline for reporting systematic reviews and meta-analyses [10] (Supplementary Material 1).

### Search process

**Figure 1.**
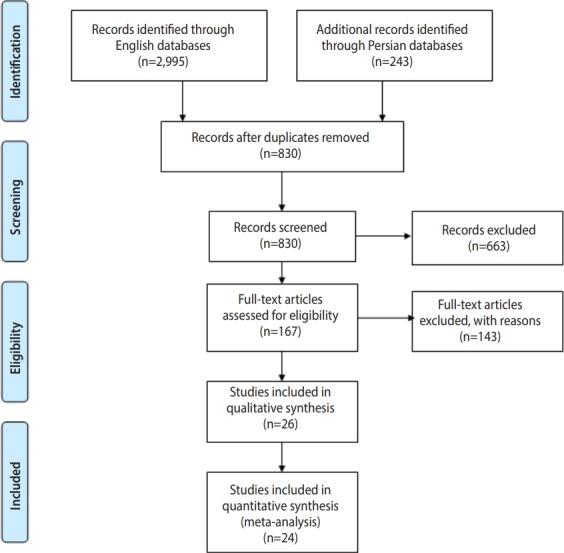
Flowchart describing the study design.

To evaluate trematode cercariae infections in snails in various regions of Iran, we carried out a systematic review of the literature (full-text) published online in the English and Persian languages. Records from 10 databases (PubMed, Science Direct, Web of Science, Scopus, EBSCO, Google Scholar, IranMedex, Magiran, Irandoc, and SID), starting from the first study available (1974) through February 2018, were searched (Figure 1).

To avoid missing any articles, the references of all papers were thoroughly checked. We searched for keywords or subject headings including snail, gastropoda, Mollusca, trematode, Trematoda, and Iran.

### Inclusion and exclusion criteria

All Persian-language and English-language studies that determined the larval stages of trematodes in snails, either microscopically or molecularly, in Iran were included in this review. Studies in which the type of snail infection, the number of infected snails, or the surveyed province was not individually considered were excluded from the review.

### Study selection

Two authors (SD and EK) independently assessed the titles and abstracts of the articles. The full texts of relevant studies were reviewed independently to ensure their eligibility for inclusion in this review. Disagreements were resolved by discussion or by a third researcher (MM).

### Quality assessment

The quality of each study was independently evaluated by 2 authors (SD and MM) according to Newcastle-Ottawa Scale [11] (Supplementary Material 2). This tool includes 3 main sections, the first section is graded on a scale containing 5 stars and mainly assesses the methodological quality of an individual study. The second section is graded from 2 stars and focuses on the comparability of the study. Finally, the third section, which is graded from 3 stars, deals with the outcomes and statistical analysis of the original study. Studies with a score of 50.0% and above were considered to have high methodological quality.

### Data extraction

Information regarding the species of snails, year of publication, areas of study, number of snails examined, number of infected snails, prevalence of infection, method of study, stage of development, parasites isolated, and first author was extracted from the articles and entered into an Excel spreadsheet (Table 1).

### Data analysis

StatsDirect statistical software (StatsDirect Ltd., Cambridge, UK) was used for data analysis. For each study, the standard deviation of the prevalence was calculated using a binomial distribution formula. The degree of heterogeneity among the results was determined based on the Q test and the I^2^ indicator. I^2^ indicates the percentage variation between studies attributed to heterogeneity compared to chance. I^2^ values ≤50.0% indicate low heterogeneity, and I^2^ values between 50.0% and 75.0% suggest moderate heterogeneity. Values >75.0% indicate high heterogeneity across studies, meaning that pooled prevalence must have considered by using a random-effects model [12]. To investigate possible sources of heterogeneity, a meta-analysis was performed on some snail species and provinces. Point estimations of the prevalence of infections with 95% confidence intervals (CIs) are shown in forest plots. The size of each square illustrates the weight of each study, while the crossed lines indicate the CIs. The bias in the results was examined by Begg and Egger tests.

## RESULTS

Of the 2,408 studies identified through the initial search, 24 were included in this systematic review, as shown in Table 1 [8,13-35]. A flowchart describing the study design is shown in Figure 1.

A total of 98,235 freshwater snails of 9 different species from the families Lymnaeidae, Physidae, Planorbidae, Viviparidae, Thiaridae, and Melanopsidae were collected and identified from different parts of Iran. The prevalence of larval trematodes in the various snail species and areas investigated is shown in Table 1. Additionally, the total quality of all primary studies was greater than 4, indicating that the quality of the included papers was medium to high.

**Figure 2.**
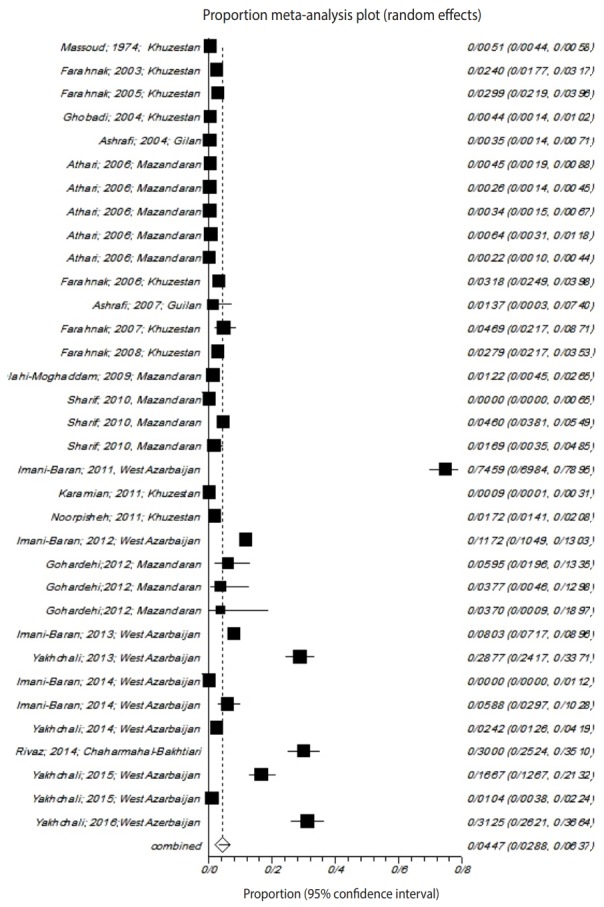
Forest plot diagram of studies showing the prevalence of trematode infections in the examined snails in Iran.

Considerable variation was present in the number of trematode cercariae-infected snails across studies; the heterogeneity was quite high (Q=4,187.3, p<0.001), and the I² obtained was 99.2% (Figure 2).

Using a random-effects model for the meta-analysis, 4.4% (95% CI, 2.8 to 6.3) of the collected gastropods were found to be infected with fluke larvae.

### Analysis of bias in the findings

**Figure 3.**
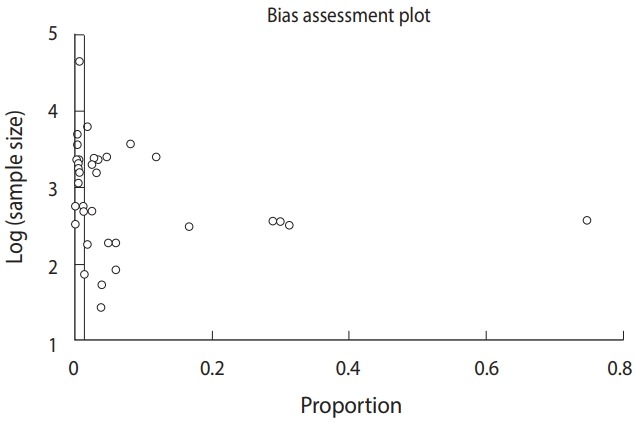
Funnel plot analysis for finding bias.

Publication bias was analyzed using an Egger funnel plot with 95% CIs. The results of the test (Egger bias: 6.3 [95% CI, 3.3 to 9.3]; p<0.001) strongly suggested publication bias (Figure 3).

### Infections in gastropods

**Figure 4.**
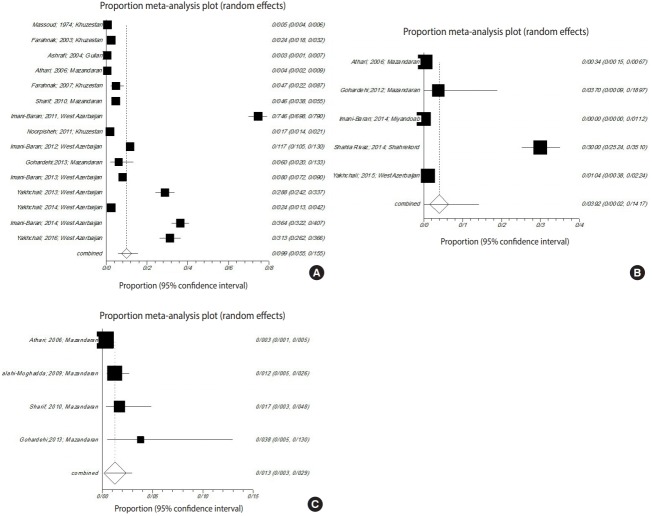
Forest plot diagram of studies showing the prevalence of trematode infections in the examined snails (A) *Radix auricularia*, (B) *Lymnaea stagnalis*, and (C) *Lymnaea palustris* in Iran.

The gastropods in all studies belonged to *Radix auricularia, Lymnaea palustris, Lymnaea truncatula, Lymnaea stagnalis, Physa gyrina spp., Planorbis planorbis, Bulinus truncatus, Bellamya (Viviparus) bengalensis, Melanoides tuberculata*, or *Melanopsis* spp. Among the snails surveyed in the meta-analysis, the prevalence of trematode infections in *R. auricularia* was 9.9% (p<0.001, Q=3,953.6), which was greater than that of *L. stagnalis* and *L. palustris*, at 3.9% (p<0.001, Q=343.9) and 1.2% (p<0.001, Q=16.8), respectively (Figure 4).

Because other infected snails were investigated in only a few studies, it was not possible to carry out a meta-analysis. Therefore, we present the frequency of infected snails with larval stages of trematodes in Table 2.

### Prevalence of larval stages of trematodes in the collected gastropods

Twelve species of trematode larvae were identified based on systematic keys, biometric examinations, experimental infections (cercariae isolated from snail were transferred into glass petri dishes containing natural water for metacercariae formation; wild-type laboratory mice were orally inoculated with metacercariae, euthanized after 8 weeks, and their livers, peritoneum walls, and abdominal cavities were checked for adult flukes), molecular examinations (polymerase chain reaction [PCR] or PCR with restriction fragment length polymorphism analysis), and staining with formaldehyde alcohol azocarmine lactophenol. Echinostomatidae cercariae (EC), xiphidiocercariae (XC), monostome cercariae (MC), Diplostomidae cercariae (DC), Paramphistomidae cercariae, Strigeidae cercariae, Clinostomidae cercariae (CC), Heterophyidae cercariae, Philophthalmidae cercaria, Cyathocotylidae cercariae, larval stages of *Fasciola* (FL), and schistosome furcocercariae (ScF) were found in different snails (Table 3).

However, in some studies, the larval stage and parasite species were not evaluated, and only trematode larva or furcocercariae were reported. Among the studies included in the meta-analysis, the highest prevalence of cercariae was found for EC and XC (4.3% and 4.1%, respectively). The prevalence of other infections is shown in Table 4.

Since the other larval stages were surveyed only in a limited number of studies, it was not possible to conduct a meta-analysis; instead, we used the average number of larval stages, as shown in Table 5.

We found that *R. auricularia* was infected with the largest number of larval trematodes (EC, XC, MC, DC, CC, FL, and ScF). Other snails found to be intermediate hosts for different cercariae are shown in Table 3.

### Prevalence of infected gastropods in different provinces of Iran

Freshwater snails were studied in 5 provinces of Iran—West Azerbaijan, Mazandaran, Guilan, Khuzestan, and Chaharmahal-Bakhtiari—that are important for agriculture.

**Figure 5.**
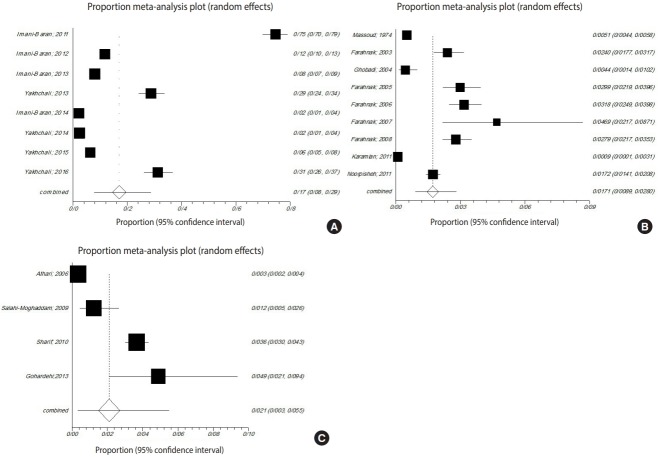
Forest plot diagram of studies showing infections of gastropods in, (A) West Azerbaijan; (B) Khuzestan; (C) Mazandaran.

In 3 provinces (Khuzestan, Mazandaran, and West Azerbaijan), studies examined the prevalence of snails infected with the larval stages of trematodes. Among these 3 provinces, West Azerbaijan showed the highest prevalence of infected snails (16.9%; 95% CI, 7.9 to 28.5; Q=1,093.6; p<0.001). In Khuzestan Province, the pooled proportion was 1.7% (95% CI, 0.8 to 2.7; Q=362.2; p<0.001). In Mazandaran Province, the pooled proportion was 2.1% (95% CI, 0.3 to 5.4; Q=208.0; p<0.001) (Figure 5).

Two studies in Guilan Province, performed by Ashrafi et al. [16,25], showed that 7 of 2,028 (2004) and 1 of 73 (2007) snails were infected with larval stages of *Fasciola gigantica* and *F. hepatica*, respectively. Furthermore, 105 of 350 snails examined by Rivaz et al. [27] in Chaharmahal-Bakhtiari Province were found to be infected with Plagiorchiidae cercariae.

## DISCUSSION

To our knowledge, this is the first systematic review of cercarial infections in snails in Iran. In this systematic review and meta-analysis, the overall prevalence of freshwater gastropods infected with cercariae in Iran was estimated to be 4.4% (95% CI, 2.8 to 6.3). The prevalence of cercarial infections in gastropods has been reported worldwide; similar studies in Turkey, Pakistan, and Iraq have reported high prevalence rates of these infections (7.3%, 14.8%, and 30.7%, respectively) [36-38]. The prevalence in countries such as Tanzania (1.3%) and France (1.9%) is lower [39,40], while the prevalence in Nepal (4.3%) is similar to that found in our study [41].

Among the snail species surveyed in this meta-analysis, *R. auricularia* (9.9%) was the most commonly infected. Some gastropods were not included in this meta-analysis because an inadequate number of articles investigated them. Since sequencing of ribosomal DNA ITS-2 demonstrated that *Lymnaea (Radix) gedrosiana*, which has been reported in many European countries [42], is a synonym of *R. auricularia* (the haplotype reported in Bandar-Anzali, northern Iran), in this study, *R. auricularia* was employed instead. Notably, the larval stages of Echinostomatidae, Monostome, Diplostomidae, Clinostomidae, Plagiorchiidae, *Fasciola*, and schistosomes were found in *R. auricularia*.

The prevalence of infections in *L. stagnalis*, which functions as a host of various trematode larvae, such as Plagiorchiidae, *Fasciola*, and schistosome larvae, was reported to be 3.9%. Soldánová et al. [43] reported that 15.1% of *L. stagnalis* samples were infected by species from the families Echinostomatidae, Diplostomidae, Plagiorchiidae, Schistosomatidae, and Telorchiidae in the Ruhr River of Germany.

Despite the broad range of data related to the prevalence of cercariae in snails, only 4 types of cercariae could be evaluated by a meta-analysis because of the insufficient number of studies on other cercariae. In this review, the prevalence of EC infection in *R. auricularia*, *M. tuberculata*, and *Melanopsis* spp. was 4.3%. This rate is lower than that reported by Abdul-Salam (6.1%) in a study on *Clypeomorus bifasciata* in Kuwait [44]. In Bangladesh, EC was also isolated from *R. auricularia* [45].

In general, the presence of EC in various snails indicates the important role of the gastropods in transmission of EC to birds, including aquatic wild birds as well as domesticated birds, in Iran and other countries. Ranjbarbahadory et al. [46], in a study on gastrointestinal helminths of native turkeys in Amol, Iran, recorded a prevalence of *Echinostoma* of 11.0%. Humans become infected by eating the infected freshwater gastropods. Echinostomiasis is endemic in East and Southeast Asia; however, Ahmadi et al. [47] recorded a 0.9% prevalence of *Echinostoma* spp. in rehabilitation centers in Mazandaran Province in northern Iran.

In this study, the prevalence of XC in various gastropod species was found to be 4.1%. Parasites isolated from *L. stagnalis, L. palustris, V. bengalensis*, and *M. tuberculata* were from the Plagiorchiidae family. In a study of freshwater resources in Poland, the Plagiorchiidae family was detected in Lymnaeid gastropods [48]. Species of *Plagiorchis* (family: Plagiorchiidae) have been reported as intestinal trematodes in birds, reptiles, and mammals. Human infections due to *Plagiorchis* spp. are rare; at present, only 11 cases have been found in humans worldwide [49]. Furthermore, XC generally belong to the Heterophidae, Opisthorchidae, and Fascolidae families, which underscores the veterinary relevance of these snails. It is very unlikely for humans to be infected by these parasites under normal conditions [50].

In Iran, especially in the coastal areas of the Caspian Sea and Persian Gulf, animal fascioliasis has been prevalent over the past 50 years. Despite the high incidence of livestock infections in the southern regions over the past decades, infections in humans have often occurred in the northern provinces, especially in Guilan Province [51]. The 2 species of *Fasciola* show variant Lymnaeid snail hosts. *F. gigantica* is transmitted by species of the *Radix* genus, while *F. hepatica* is mostly transmitted by species of the *Galba/Fossaria* genera [42]. In France, *L. neotropica, L. viatrix* var. *ventricosa*, and *Galba truncatula* are known to be hosts of *F. hepatica* [52]. It is noteworthy that human fascioliasis has emerged as a public health problem in Kermanshah Province in western Iran; hence, the verification of new regions of human fascioliasis requires complementary investigations [53].

Both mammalian and bird schistosomes are causative agents of human cercarial dermatitis, but bird schistosomes, mostly *Trichobilharzia* species, are responsible for the majority of dermatitis outbreaks reported both in Iran and worldwide [13,54]. Recently, in a study conducted in Mazandaran Province, Iran in 2016, Fakhar et al. [55] reported that all the examined samples of nasal schistosomes were grouped in a sister clade to the European *Trichobilharzia regenti*.

In Mazandaran Province, Gohardehi et al. [13] showed that the prevalence of infections with *Trichobilharzia* spp. among migratory birds (ducks) was 15.8% and the most infected snail was *R. auricularia*. In addition, in 2013, Rahimi-Esboei et al. [56] reported a high prevalence (77.5%) of cercarial dermatitis among paddy field workers in Mazandaran Province, thereby proving it to be a health hazard in the area. In this review, schistosome furcocercariae were detected in 5 snail species: *R. auricularia, L. palustris, L. stagnalis, P. planorbis*, and *M. tuberculata*, with a prevalence of 2.7%. The prevalence of furcocercariae in *Bulinus globosus* (1.1%) in Tanzania [39] was less than that found in our study. *Bulinus* spp. act as intermediate hosts in the life cycle of *S. haematobium*; despite their remarkable abundance, only a single study, by Farahnak et al. [28], has surveyed infections by trematodes in *Bulinus* spp. [57].

In this study, different cercariae were detected in several provinces of Iran because of variations in rainfall, humidity, and temperature in each province. Among the provinces evaluated in this meta-analysis, the highest and lowest proportions of cercarial infections were observed in West Azerbaijan (16.9%) and Khuzestan (1.7%) Provinces, respectively. Temperatures of 25-30°C, annual rainfall exceeding 100 mm, and relative humidity >65.0% are favorable for the growth and shedding of cercariae [58]. These conditions facilitate their development and growth in host snails, and they change from miracidia to sporocysts, rediae, and eventually cercariae. The climate in West Azerbaijan is mainly influenced by the rain-bearing winds of the Mediterranean Sea and Atlantic Ocean [59]. The mean temperature ranges from 13.7°C (winter) to 22.5°C (summer), with an average annual rainfall of 300 mm-800 mm that occurs in 2 primary rainy seasons (March to June and October to November) and a humidity of 30-80%, indicating that it has a moderate climate. Additionally, West Azerbaijan contains 30 permanent and seasonal wetlands and many suitable habitats for birds. This province is also geographically positioned along the migration path of birds that migrate from northern latitudes to southern latitudes every year at the beginning of the autumn and cold seasons. In previous studies in the province, snails were collected from May through November. The most prevalent cercarial types in the province, according to the reports by Imani-Baran et al. [33] and Yakhchali et al. [35], were EC (276 of 370), *T. szidati*, and *T. franki* (100 of 320) from *R. auricularia*, respectively. Thus, contamination by these two trematodes may be significant in West Azerbaijan. In Chaharmahal-Bakhtiari, 105 of 350 snails surveyed by Rivaz et al. [27] were infected with Plagiorchiidae cercariae.

Some limitations should be considered when interpreting the results of our study, including publication bias. The present meta-analysis only included published studies; we did not search for unpublished studies or original data. Thus, the findings of the Egger funnel plot should be interpreted with caution, because of the few numbers of included studies in combination with the high heterogeneity across the studies, which could limit the ability to assess publication bias.

Few studies have been conducted in Iran, compared to other parts of the world, of the distribution of types of snails and the prevalence of trematode infections in intermediate hosts using conventional microscopic and molecular methods. Therefore, due to the limited number of studies carried out in 5 provinces, we could not conduct a meta-analysis of all surveyed provinces, snails, and trematode infections.

## CONCLUSION

This is the first study to provide information on the distribution of various snails, their trematode infections, and their potential to cause zoonotic diseases in Iran. According to the present review, snail control is essential for reducing the prevalence of diseases such as echinostomiasis, fascioliasis, and cercarial dermatitis in humans. Implementation of snail control depends on several factors, such as the infection level in the final hosts (domestic animals or people), the freshwater snail habitat, transmission pattern, snail species, and ecological concerns.

Future studies are needed to characterize the prevalence of trematodes in snails in different provinces of Iran. It is also recommended that more accurate methods should be used for identifying cercariae at the species level, to promote a better understanding of the epidemiological conditions of these infections in different provinces.

## Figures and Tables

**Table 1.** Basic characteristics of the included studies

Author, year [Ref]	Snail species	Study areas	Collected snails (n)	Examined snails (n)	Infected snails (n)	Prevalence (%)	Study method	Developmental stage	Isolated parasite	Quality score
Massoud, 1974 [14]	*R. auricularia*	Khuzestan	44,317	44,317	225	0.5	Experimental infections	Cercariae	*O. turkestanicum*	5
Farahnak et al., 2003 [15]	*R. auricularia*	Khuzestan	2,000	2,000	48	2.4	Systematic key	Furcocercariae of avian schistosomes	*Trichobilharzia* spp.	4
Ashrafi et al., 2004 [16]	*R. auricularia*	Guilan	4,830	2,028	7	0.3	Experimental infections	Immature rediae and cercariae	*F. gigantica*	
Athari et al., 2006 [34]	*R. auricularia*	Mazandaran	14,190	1,794	8	0.4	Experimental infections	Furcocercariae of avian schistosomes, other furcocercariae	-	4
Farahnak et al., 2007 [17]	*R. auricularia*	Khuzestan	192	192	9	4.0	Systematic key	Furcocercous cercariae	Bird schistosome (*Gigantobilharzia* spp.)	4
Sharif et al., 2010 [24]	*R. auricularia*	Mazandaran	3,266	2,523	116	4.6	Systematic key	Echinostomatidae cercaria, Plagiorchiidae cercaria, Diplostomidae cercariae, Clinostomidae cercariae	-	
Noorpisheh et al., 2011 [19]	*R. auricularia*	Khuzestan	6,213	6,213	107	5.0	Systematic key	Larval stages	Trematodes	6
Imani-Baran et al., 2011 [33]	*R. auricularia*	West Azerbaijan	6,759	370	276	74.6	Systematic key	Echinostome cercariae, Furcocercariae	-	5
Imani-Baran et al., 2012 [20]	*R. auricularia*	West Azerbaijan	6,759	2,543	298	11.7	PCR	Larval stages	*F. gigantica*	6
Gohardehi et al., 2013 [13]	*R. auricularia*	Mazandaran	676	84	5	5.9	Systematic key	Furcocercariae of avian schistosomes	*Trichobilharzia* spp.	4
Yakhchali et al., 2013 [32]	*R. auricularia*	West Azerbaijan	6,759	365	105	28.8	Molecular examination	Larval stages	*O. turkestanicum*	6
Imani-Baran et al., 2013 [21]	*R. auricularia*	West Azerbaijan	6,759	3,673	295	8.0	Systematic key, biometrical examinations	Xiphidiocercariae, Furcocercariae, Echinostome cercariae, Monostome cercariae	-	6
Yakhchali et al., 2014 [9]	*R. auricularia*	West Azerbaijan	6,759	496	12	2.4	Systematic key	Larval stages	*F. gigantica*	6
Imani-Baran et al., 2014 [22]	*R. auricularia*	West Azerbaijan	514	187	11	5.9	Systematic key	Furcocercariae, Echinostome cercariae	-	5
Yakhchali et al., 2016 [35]	*R. auricularia*	West Azerbaijan	320	320	100	31.2	PCR	Larval stages	*T. szidati* and *T. franki*	6
Athari et al., 2006 [34]	*L. palustris*	Mazandaran	14,190	4,934	13	0.3	Experimental infections	Furcocercariae of avian schistosomes, other furcocercariae	*Trichobilharzia* spp.	4
Salahi-Moghaddam et al., 2009 [23]	*L. palustris*	Mazandaran	490	490	6	1.2	Systematic key	*Echinostoma* cercariae	-	7
Sharif et al. 2010 [24]	*L. palustris*	Mazandaran	3,266	178	3	1.7	Systematic key	Plagiorchiidae cercariae	-	6
Gohardehi et al., 2013 [13]	*L. palustris*	Mazandaran	676	53	2	3.8	Systematic key	Furcocercariae of avian schistosomes	-	4
Ashrafi et al., 2007 [25]	*L. truncatula*	Guilan	200	73	1	1.4	PCR	Larval stages	*F. hepatica*	4
Sharif et al., 2010 [24]	*L. truncatula*	Mazandaran	3,266	565	0	0.0	Systematic key	-	-	6
Yakhchali et al., 2015 [26]	*L. truncatula*	West Azerbaijan	6,759	306	51	16.6	PCR-RFLP	Larval stages	*F. hepatica*	6
Athari et al., 2006 [34]	*L. stagnalis*	Mazandaran	14,190	2,350	8	0.3	Experimental infections	Furcocercariae of avian schistosomes, other furcocercariae	*Trichobilharzia* spp.	4
Gohardehi et al., 2013 [13]	*L. stagnalis*	Mazandaran	676	27	1	3.7	Systematic key	Furcocercariae of avian schistosomes	*Trichobilharzia* spp.	4
Rivaz et al., 2014 [27]	*L. stagnalis*	Chaharmahal-Bakhtiari	400	350	105	30.0	Systematic key	Plagiorchiidae cercariae	*Opisthioglyphe, Plagiorchiidae*	4
Imani-Baran 2014 [22]	*L. stagnalis*	West Azerbaijan	-	327	0	0.0	Systematic key	-	-	5
Yakhchali et al., 2015 [26]	*L. stagnalis*	West Azerbaijan	6,759	579	6	1.1	PCR-RFLP	Larval stages	*F. hepatica*	6
Athari et al., 2006 [34]	*Physa gyrina *spp	Mazandaran	14,190	3,560	8	0.2	Experimental infections	Furcocercariae of avian schistosomes, other furcocercariae	-	4
Athari et al., 2006 [34]	*P. planorbis*	Mazandaran	14,190	1,552	10	0.6	Experimental infections	Furcocercariae of avian schistosomes, other furcocercariae	*Trichobilharzia* spp.	4
Farahnak et al., 2008 [28]	*B. truncatus*	Khuzestan	2,400	2,400	67	2.8	Systematic key	Amphistome cercariae, Strigea cercariae	Paramphistomidae (52)^1^, Strigeidae or Diplostomidae (15)^1^	4
Ghobadi & Farahnak 2004 [29]	*V. bengalensis*	Khuzestan	1,143	1,143	5	0.4	Systematic key	Xiphidiocercariae	Plagiorchiidae	4
Farahnak et al., 2005 [30]	*M. tuberculata*	Khuzestan	1,540	1,540	46	2.9	Systematic key, experimental infections	Heterophyidae cercariae, Echinostomatidae cercariae (*E. milvi*), Schistosomatidae, Furcocercariae, Plagiorchiidae cercariae, Philophtalmidae cercariae	Heterophyidae (26)^1^: *H. pumilio, H. taithui Stellantchasmus falcatus, Centrocestus formosanus* Echinostomatidae (1)^1^: *E. milvi* Schistosomatidae (5)^1^ Plagiorchiidae (10)^1^ Philophtalmidae (4)^1^	4
Karamian et al., 2011 [18]	*M. tuberculata*	Khuzestan	3,830	2,294	2	0.1	Molecular, staining with FAAL	Furcocercariae of schistosomes	*Gigantobilharzia–Dendritobilharzia*	4
Farahnak et al., 2006 [31]	*Melanopsis* spp.	Khuzestan	2,266	2,266	72	3.1	Experimental infections	Heterophyidae cercariae: (*H., pumilio, H. taithui, Stellantchasmus falcatus, Centrocestus formosanus*)	-	4
								Echinostome cercariae (*E. milvi*)		
								*Cyathocotylid* cercariae		
								Philophthalmidae cercariae		
								Monostome cercariae		

*R., Radix; L, Lymnaea; P., Planorbis; B., Bulinus; V., Viviparus; M., Melanoides; H., Haplorchis; E., Echinochasmus; F., Fasciola; O., Ornithobilharzia; T., Trichobilharzia*; PCR, polymerase chain reaction; RFLP, restriction fragment length polymorphism; FFAAL, formaldehyde alcohol azocarmine actophenol.

1 Number infected snails with the trematode larvae.

**Table 2.** Frequency of infections of snails with larval stages of trematodes in Iran during 1974-2018

Snail types	Studies (n)	Examined snails (n)	Infected snails (n)	Infected snails(%)
*Lymnae truncatula*	3	944	56	5.9
*Physa gyrina* spp.	1	3,560	8	0.2
*Planorbis planorbis*	1	1,552	10	0.6
*Viviparus bengalensis*	1	1,143	5	0.4
*Bulinus truncatus*	1	2,400	67	2.8
*Melanoides tuberculata*	2	3,834	48	1.2
*Melanopsis* spp.	1	2,266	72	5.9

**Table 3.** Snail species infected with various larval trematodestages in Iran

Snail types	No. of cases infected with various larval trematode stages														
	Examined individuals (n)	EC	XC	MC	DC	CC	HC	PhC	CyC	FL	ScF	PC	SC	TL	FC
*R. auricularia*	106,090	283	271	4	84	2	-	-	-	317	495	-	-	107	120
*L. palustris*	5,655	-	3	-	-	-	-	-	-	-	11	-	-	-	10
*L. truncatula*	944	-	-	-	-	-	-	-	-	52	-	-	-	-	-
*L. stagnalis*	3,633	-	105	-	-	-	-	-	-	6	2	-	-	-	7
*Physa gyrina* spp.	3,560	-	-	-	-	-	-	-	-	-	-	-	-	-	8
*P. planorbis*	1,552	-	-	-	-	-	-	-	-	-	1	-	-	-	9
*B. truncatus*	2,400	-	-	-	-	-	-	-	-	-	-	52	15	-	-
*V. bengalensis*	1,143	-	5	-	-	-	-	-	-	-	-	-	-	-	-
*M. tuberculata*	3,834	1	10	-	-	-	26	4	-	-	7	-	-	-	-
*Melanopsis* spp.	2,266	2	-	3	-	-	43	5	19	-	-	-	-	-	-
Snails examined and infected with larval stages	131,077	286	394	7	84	2	69	9	19	375	516	52	15	107	154

*R., Radix; L., Lymnaea; P., Planorbis; B., Bulinus; V., Viviparus; M., Melanoides*; EC, Echinostomatidae cercariae; XC, xiphidiocercariae; MC, monostomecercariae; DC, Diplostomidae cercariae; CC, Clinostomidae cercariae; HC, Heterophyidae cercariae; PhC, Philophthalmidae cercariae; CyC, Cyathocotylidae cercariae; FL, larval stages of *Fasciola*; ScF, schistosome furcocercariae; PC, Paramphistomidae cercariae; SC, strigeacercariae; TL, non-identified trematode larvae; FC, non-identified furcocercariae.

**Table 4.** Data related to forest plot diagrams of studies showing the prevalence of trematode cercariae in Iran

Trematode larval stages	Pooled proportion (%)	I² (inconsistency), % (95% CI)	Cochran Q	df	p-value
EC	4.3	99.6 (99.6, 99.7)	1,357.0	5	<0.001
XC	4.1	99.0 (98.8, 99.1)	485.7	5	<0.001
FL	3.7	99.0 (98.7, 99.2)	390.8	4	<0.001
FC	2.8	97.2 (96.0, 97.9)	143.7	4	<0.001
ScF	2.7	98.4 (98.2, 98.6)	898.1	14	<0.001

CI, confidence interval; EC, Echinostomatidae cercariae; XC, xiphidiocercariae; FL, larval stages of Fasciola; FC, non-identified furcocercariae; ScF, schistosome furcocercariae.

**Table 5.** Average number of larval stages isolated from snails in Iran

Larval trematode stages	Studies (n)	Examined larval stages (n)	Identified larval stages (n)
MC	2	5,939	7
DC	1	2,523	84
CC	1	2,523	2
HC	2	3,806	69
PhC	2	3,806	9
CyC	1	2,266	19
TL	1	6,213	107
PC	1	2,400	52
SC	1	2,400	15

MC, monostome cercariae; DC, Diplostomidae cercariae; CC, Clinostomidae cercariae; HC, Heterophyidae cercariae; PhC, Philophthalmidae cercariae; CyC, Cyathocotylidae cercariae; TL, non-identified trematode larvae; PC, Paramphistomidae cercariae; SC, strigea cercariae.
